# Distribution and seasonal abundance of medically important flies in Sharkia Governorate, Egypt and their associated bacteria

**DOI:** 10.1371/journal.pone.0348022

**Published:** 2026-05-04

**Authors:** Tharwat A. Selim, Nosiba S. Basher, Saber A. Riad, Ahmed M. Al-Shahat, Amr Hosny Hashem, Randa I. Eltaly, Enayat M. Elqady, Eman El-said, Fatma Z. Hamed, Naglaa Fathi Badr, Zeinab A. Shouaib, Mohammed E. Rashed, Nasir A. Ibrahim, Sulaiman A. Alsalamah, Fahd A. Nasr, Mohamed A. M. El-Tabakh

**Affiliations:** 1 Department of Zoology and Entomology, Faculty of Science (Boys), Al-Azhar University, Nasr City, Cairo, Egypt; 2 Biology Department, College of Science, Imam Mohammad Ibn Saud Islamic University (IMSIU), Riyadh, Saudi Arabia; 3 Botany and Microbiology Department, Faculty of Science, Al-Azhar University, Cairo, Egypt; 4 Department of Zoology and Entomology, Faculty of Science (Girls), Al-Azhar University, Nasr City, Cairo, Egypt; 5 Research Institute of Medical Entomology, Ministry of Health and Population, Dokki, Giza, Egypt; National Research Centre, EGYPT

## Abstract

Due to the medical importance of flies as mechanical vectors of numerous pathogens, accurate information on their distribution, abundance, and associated bacterial communities is essential. This study investigated the diversity, preliminary seasonal observations, and bacterial associations of medically important flies in Sharkia Governorate through field surveys conducted from 2022 to 2023. A total of twelve fly species belonging to five families Calliphoridae, Muscidae, Sarcophagidae, Piophilidae, and Phoridae were identified. *Chrysomya megacephala* exhibited marked seasonal variation, whereas *Sarcophaga carnaria* showed relatively stable activity. *Chrysomya albiceps*, *Lucilia sericata*, and *Piophila casei* were absent during winter despite their presence in summer. *Musca domestica* was the most abundant species across all seasons, with Muscidae representing the dominant family (p < 0.001), followed by Calliphoridae (p < 0.05), while other families were significantly less abundant. Biodiversity indices, including Shannon and Simpson metrics, indicated high species diversity throughout the year with a slight decline during winter. Evenness values reflected balanced species distribution, and the highest Fisher’s alpha and Margalef richness indices were recorded during summer, highlighting the influence of temperature on community structure. Bacterial analysis of *M. domestica* body surfaces revealed that 80% of isolates were pathogenic species, while 20% were classified as non-pathogenic. These findings emphasize the ecological and public health significance of flies and highlight their potential role in pathogen transmission within the study area.

## Introduction

The order Diptera represents an ecologically and medically important group of insects, as many of its species possess significant medical, agricultural, and veterinary relevance [1,2]. Among dipterans, flies are particularly widespread due to their close association with human habitats [3], where they cause considerable nuisance and contribute to disease transmission through contact with human bodies and food sources [4,5]. In addition, several fly species are of veterinary concern because they can induce myiasis in animals [6]. Flies occur worldwide across nearly all ecosystems and typically feed on organic materials in liquid or semi-liquid forms. Consequently, they have been implicated in the transmission of numerous diseases; for example, *Musca domestica* is recognized as a mechanical vector of more than 65 human diseases, including giardiasis and amebiasis [7].

Furthermore, some dipterous taxa are recognized for their economic significance because of their high density, which is linked to financial losses in farms that produce eggs and poultry since it disrupts workers and lowers the quality of the final products [8,9]. The expense of controlling flies in chicken production farms is well known. For instance, the United States spends over two million dollars a year to manage the number of flies in poultry farms and residential areas [10]. Examining the prevalence, range, and variety of dipterous flies in both urban and rural settings is one of the most significant health concerns [11–13]. Furthermore, surveying the flies’ distribution, prevalence, and diversity is essential to a successful management program since this biological data will enhance the effectiveness of the methods used nowadays [4,14].

Dipteran flies are strongly associated with disease outbreaks and economic losses due to their ability to contaminate food and disseminate pathogens through their sponging mouthparts, regurgitation behavior, body hairs, adhesive foot structures, and digestive systems [15–17]. Adult flies frequently inhabit microbe-rich environments such as animal waste, manure, decomposing organic matter, and spoiled food sources, which enhances their capacity to acquire and transmit microorganisms [18,19].

Additionally, studies on the microbiome of dipteran flies advance our knowledge of the pathogenic agents that house flies encounter and the ways in which they transmit disease [20]. Research has identified a broad range of bacteria, including both Gram-positive and Gram-negative species, that have been isolated from insect surfaces [21]. *Bacillus, Pseudomonas, Staphylococcus, Enterobacter,* and *Serratia* are among the genera of bacteria that are frequently isolated [22]. In insect biology, these bacteria may play a number of roles, including communication, pathogen defense, and food provisioning [23]. Despite the recognized medical importance of flies as mechanical vectors of numerous pathogenic microorganisms, comprehensive ecological data on their species composition, seasonal dynamics, and distribution patterns remain limited in many regions of Egypt, particularly in Sharkia Governorate. Previous studies have largely focused on individual species or short-term surveys, with insufficient integration of biodiversity assessment and seasonal variation analysis. Therefore, there is a significant lack of updated, region-specific information linking fly diversity, abundance patterns, and their potential epidemiological importance, highlighting the need for systematic field investigations to better understand their ecological distribution and public health implications.The aim of this study was to investigate the distribution, abundance, and diversity of medically important fly species in different locations of Sharkia Governorate. Additionally, the study sought to provide accurate information on the seasonal dynamics of these flies and their potential role as carriers of associated pathogenic bacteria.

## Materials and methods

### Study area

**Fig 1:**
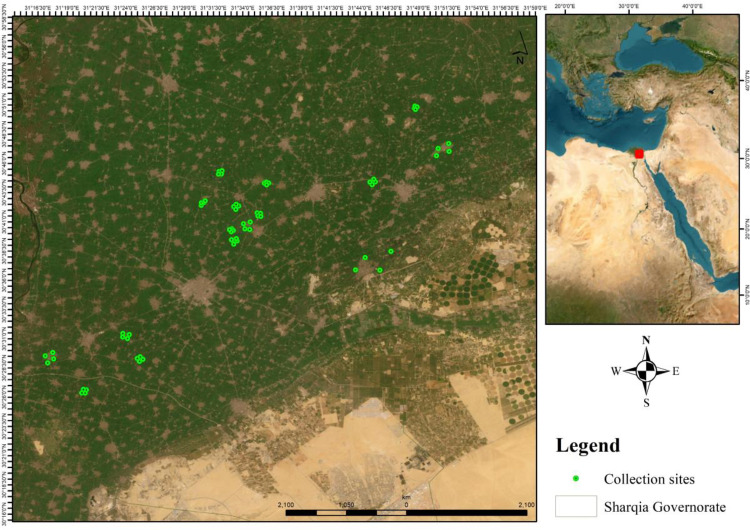
Basemap source: USGS National map viewer.

El Sharqia Governorate is located in the northern Nile Delta of Egypt, spanning approximately between longitudes 31° 15′ and 32° 15′ E and latitudes 30° 20′ and 31° N. With a territory of 4,922 km^2^. To know the distribution and diversity, Seasonal Abundance, and Bacterial Pathogens of Medically Important Flies in Sharkia Governorate, Egypt, four stations were chosen to cover most of the governorate. Each station was divided into four sites, and each site contained four points from which species was collected as follow: {Faqus (Al. Nahaseen, Al. Samanh, AL. Fadadnh, Al. gaafrh); Hehia (Kafr.abo.Hatb, Mahdia, Al.ehsania, AL.shebrawean); Al.Ibrahimia (Kofor.negm, Tal.mohamed, Al.halawat, Mubasher); Minya Al Qamh (Meet- bashar, Shubra- alaneb, Al-azezia, Bany.helal)} Fig 1.

### Field trips and sample collection

Over the course of a year, from 2022 to 2023, with average temperatures and humidities during four seasons [summer (31.2 °C, 50%), autumn (27.5 °C, 48%), winter (17.8 °C, 45%) and spring (23.3 °C, 45%)], respectively, flies were surveyed on multiple field visits at various carefully chosen stations throughout the Sharqia Governorate. With minor adjustments, we employed typical baited traps from study [24] for our investigation. Fish (50g per trap) were employed as bait in these traps. Each study location had a single trap set up for the 24-hour fly collection period. Traps were put up for the day’s collection between 11:00 and 11:30 am, and they were collected simultaneously on the second day. At every study location, four replicates were carried out.

### Identification of species

Samples were examined and separated from the baits and transferred to the laboratory to complete the separation and identification processes. After collection, adult flies were preserved in vials labeled with date and time of collection. Collected insects were identified to species by using different keys **[**25–27**].**

### Isolation and identification of associated bacteria

Different types of enrichment and selective media were utilized for the isolation and preliminary identification of bacterial isolates obtained from four collected samples. The media employed included Blood agar, MacConkey agar, Nutrient agar, Mannitol Salt Agar (MSA), and Eosin Methylene Blue (EMB) agar/ Cystine Lactose Electrolyte Deficient (CLED) agar. Following isolation, the colonies were purified through successive subculturing, and the isolates were subjected to preliminary characterization based on Gram staining and morphological appearance. Subsequently, bacterial identification was confirmed using the VITEK automated system, which applies a series of biochemical tests such as Catalase, Coagulase, Oxidase, Urease, Indole, Citrate, ONPG (β-galactosidase), Hydrogen sulfide (H₂S), Gelatinase, and DNase. The obtained biochemical profiles were analyzed, and each isolate was identified to the species level with corresponding probability percentages [28].

### Data analysis

The statistical software SPSS V.22 was used to code and input the data. Data were tested for satisfying assumptions of parametric tests; continuous variables were subjected to Shapiro-Wilk and Kolmogorov-Smirnov tests for normality. Probability and percentile data were standardized for normality using Arcsine Square Root. Data was presented as Median and SE of Median regarding the abundance and mean ± standard deviation regarding calculated Environmental indices. ANOVA analyses were done for the investigated sites regarding the recorded insects abundance; analysis was evaluated using at least three replicates of traps at each site; post-hoc analysis was assessed using Fisher LSD pairwise comparison using MiniTab V 14; P-values were considered significant at<0.05. 2. Data were visualized when possible, using R studio V 2022.02.4.

The following biodiversity indices were applied to evaluate species diversity and community structure across the investigated study sites. Each index was calculated according to standard ecological formulas, as described below.

1. Simpson’s Index (1-D)

Simpson’s Index (1 − D) was used to assess community diversity by incorporating both species richness and species evenness. The index is calculated using the following equation:


𝐃=1−∑(𝐧_𝐢(𝐧_𝐢−1)/𝐍(𝐍−1))

where $n_{i}$represents the number of individuals belonging to species i, and Ndenotes the total number of individuals within the community.

3. Shannon Index (H)

The Shannon diversity index was employed to estimate community entropy, reflecting both species richness and the distribution of individuals among species. It is expressed as:


𝐇'=−∑(𝐩_𝐢*𝐥𝐧𝐩_𝐢)

where p_i is the proportion of individuals in species i.

4. Menhinick Index

The Menhinick Index measures species richness relative to the square root of the total number of individuals. It is calculated as:


𝐌=𝐒/𝐍

where S is the number of species, and N is the total number of individuals.

5. Margalef Index

The Margalef Index quantifies species richness adjusted for sample size. It is given by:


𝐃=(𝐒−1)/𝐥𝐧𝐍

where S is the number of species, and N is the total number of individuals.

6. Individuals (N)

The total number of individuals in the community, N, was counted as the sum of individuals across all species:


𝐍=∑𝐧_𝐢

where n_i represents the number of individuals of species i.

7. Fisher’s Alpha

Fisher’s Alpha is an index used to estimate the number of species based on the number of rare species. The formula is:


α=(𝐒−1)/(𝐥𝐧(𝐍)−𝐥𝐧(𝐍−𝐒))

where S is the number of species, and N is the total number of individuals.

8. Evenness (e^H/S)

The Evenness Index, based on the Shannon Index, measures how evenly individuals are distributed across species. The formula is:


𝐄=𝐞^𝐇'/𝐒

where H’ is the Shannon Index, and S is the number of species.

9. Equitability (J)

Equitability measures the evenness of species abundance in the community and is calculated as:


𝐉=𝐇'/𝐥𝐧𝐒

where H’ is the Shannon Index, and S is the number of species.

10. Dominance (D)

Dominance quantifies the extent to which a few species dominate the community. It is calculated using the formula:


𝐃=∑𝐩_𝐢^2

where p_i is the proportion of individuals of species i.

11. Brillouin Index

The Brillouin Index measures biodiversity while accounting for both species abundance and number. The formula is:


H_B=(1/ N)*ln(N!/∏(i=1 to S) n_i!)

where N is the total number of individuals, S is the number of species, and n_i is the number of individuals of species i.

12. Berger-Parker Index

The Berger-Parker Index is a measure of dominance, calculated as the proportion of the most abundant species relative to the total number of individuals:


𝐁𝐏=𝐧_𝐦𝐚𝐱/𝐍

where n_max is the number of individuals in the most abundant species, and N is the total number of individuals.

## Results

### The spatial and temporal abundance of insect’s species and families at investigated sites

**Fig 2:**
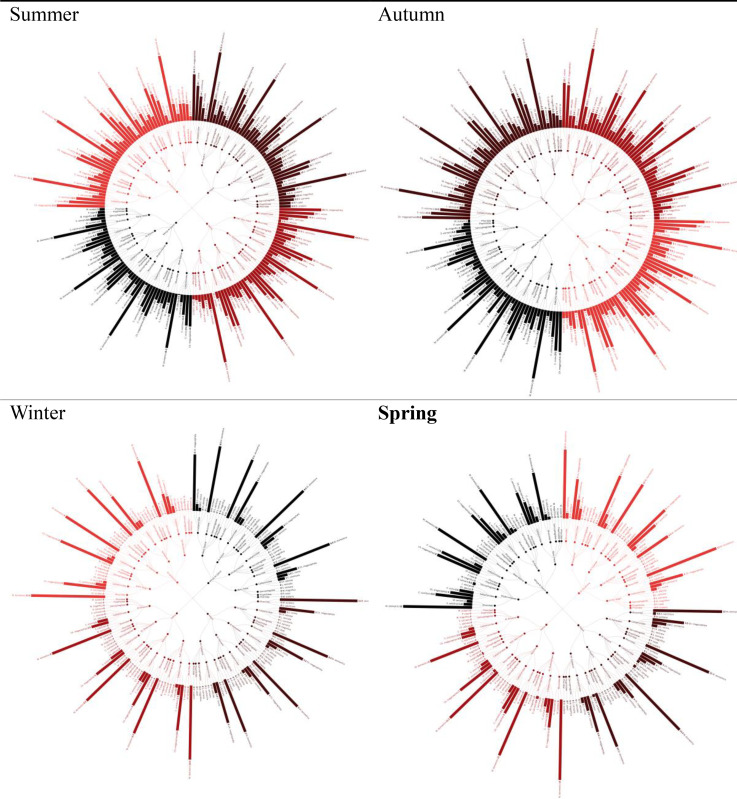
Radial bar tree represents the spatial and temporal abundance of insects at investigated sites.

In the present results, twelve fly’s species belonging to five families namely, Calliphoridae (*Chrysomya albicep, Chrysomya megacephala, Lucilia sericata, Calliphora vicina, Calliphora vomitoria*), Muscidae (*Musca domestica.Musca sorbens, Stomoxys calcitrans*), Sarcophagidae (*Sarcophaga carnaria, Wohlfartia magnifica*), Piophilidae (*Piophila casei*) and Phoridae (*Megaselia scalaris*) were inhabiting Sharkia Governorate. There were particular seasonal and taxonomic trends of dipteran abundance as well as an observable pattern of spatial distributions of the surveyed governorate. There were considerable changes in population densities (p < 0.05). When compared to the fall population, the summer populations were the largest, with the number of individuals per sampling site reaching their highest value considerably (P < 0.05). There were significantly low winter (P < 0.05) and spring (P < 0.05) population with no significant intersessional variation (P > 0.05). This tendency was more pronounced in calliphorids, where *Chrysomya albiceps* and *Lucilia sericata* had disappeared during winter season, whereas *Musca domestica* still retained the traces of population in wintertime and proved to be the most abundant species during all seasons. (P < 0.05). Spatially there was no significant difference in abundance across governorate (p > 0.05 in all seasonal comparisons). Non-significant trends however implied the presence of climatic factors, where both Al-Ibrahimia and Faqus recorded slightly greater numbers during summer compared to Minya Al-Qamh (P < 0.05). These trends were estimated to be similar in all taxonomic groups, suggesting that there were broadscale environmental factors dominating local habitat variation during different seasons. Spectacular seasonal variation of *Chrysomya megacephala* with moderate but constant activity of *Sarcophaga carnaria* might be seen. (S1–S4 Tables and Fig 2).

**Fig 3:**
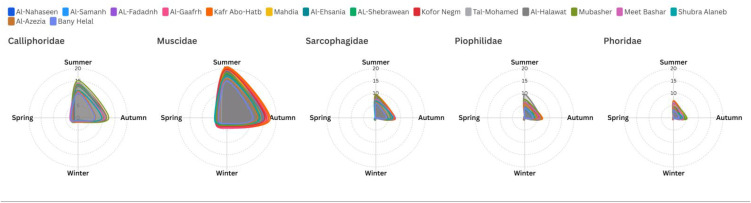
Radar chart represents the spatial and temporal abundance of insect’s families at investigated sites.

There were noticeable seasonal differences (P < 0.05) in the distribution patterns of dipteran families, but much less geographic site to site differences (P > 0.05). Calliphoridae presented well-evident seasonal trends, being abundant during summer and autumn months and then falling off dramatically during winter and only partially recovering during spring (P < 0.05). The most ecologically resistant family was seen to be Muscidae since it had large populations all year round (P > 0.05). Summer populations were at peak level, and the family exhibited high resistance in lower temperatures than other families of dipterans. This synchronicity in the two seasons, and two geographical locations highlights how extraordinary the family adjusts to the different environmental circumstances. Sarcophagidae were intermediate as regards the abundance pattern with less seasonal variation than Calliphoridae. When it comes to summer and autumn, the family managed to keep detectable populations, yet it became nearly nonexistent during winter seasons (P < 0.05). The spring re-occurrence of the family was always confined in all places implying similar emergence timings tethered to temperature levels. Piophilidae and Phoridae had the most limited seasonal behavior with none performing in the winter months and the fewest in spring. Both of the families experienced their peak abundance in summer with a slightly better persistence of Piophilidae in autumn than Phoridae. The spatial pattern of these families was especially homogeneous, and no location was supporting appreciably more or less population than other locations at high seasons (Fig 3).

### The diversity indices at investigated sites

**Fig 4:**
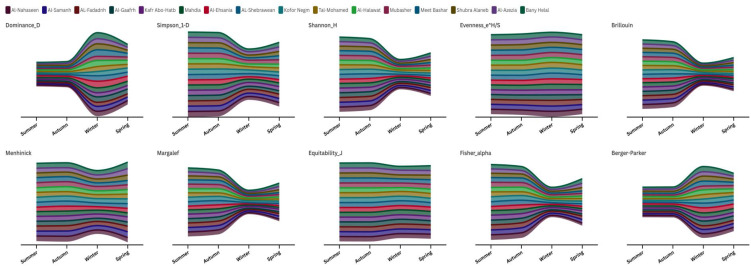
Stream graph represents the Environmental indices at investigated sites.

Shannon_H values and Simpson_1-D indices showed high levels of diversity all through the year, although with a small drop during winter months, showed that all the sites had well balanced communities that contained multiple species that did not have strong dominance. Conditions during this period were also fairly stable and evenness indices (Equitability_J) reflect distributed resources among species evenly. Summer had the highest alpha values of Fisher= and Margalef and indicatedg that species richness is optimal at this time of the year, probably due to high temperatures and availability of resources that increased the carrying capacity of the habitat. While Autumn was an intermediate period in which the diversity metrics were not very low yet started to indicate reorganization of the community. Winter also resulted in a drastic change in the structure of communities making all of the indices highly significant (p < 0.05). Berger-Parker index increased as new hierarchies of dominance were formed with the loss of cold-intolerant species. Simultaneously, the Simpson 1-D index and Shannon-H fell, indicating a significant loss of diversity, while spring brought the start of community recovery, but the process showed insignificant (P > 0.05) progress. The diversity indices were more or less in the middle, with the Shannon_H and Simpson_1-D being significantly lower (P < 0.05) than during summer-autumn but higher than during winter. Fisher alphas were slow to recover against abundance patterns, with Menhinick index indicating rapid recolonization. (S5 Table & Fig 4). The spatial analysis indicated rather unexpected consistency between governorate, where the majority of indices depicted no significant variances (p > 0.05) between sites. Faqus showed higher Fisher alpha and Margalef values than the other locations, indicating a modest regional pattern in the measures related to richness. The inverse relationship between Dominance_D and Simpson_1-D indices were statistically consistent in all seasons and sites and therefore, justified their value as complementary measurements in the ecosystem. The extreme values of winter showed how environmental stress destroys other community structures, whereas, in summer optimal conditions allowed complex networks to exist with ecological interactions. The transitional values of Spring were especially enlightening as they showed that reassembling of the community is predictable with positive conditions (S5 Table & Fig 4). The combination of results gives a description that ecosystems are very seasonal and fluctuations in temperature are a master regulator of community structure. Such persistence on the part of these communities, especially, their responsiveness to a diversity increase in spring, indicates adjusted adaptations to regular yearly changes.

### Isolation and identification of associated bacteria

The biochemical and morphological characteristics of the bacterial isolates obtained from various sources are summarized in Table 1. Most isolates were Gram-negative bacilli, while a few were Gram-positive cocci. Catalase activity was positive in all isolates, indicating their ability to decompose hydrogen peroxide. Variations were observed in other biochemical tests such as urease, oxidase, citrate utilization, gelatinase, and DNase, which are useful for differentiating species within and between genera. Growth patterns on selective and differential media (e.g., MacConkey agar, MSA, and EMB/CLED) further aided in the preliminary identification of each isolate.

**Table 1:** Morphological and Biochemical Properties of the Bacterial Isolates.

Catalase	Coagulase	Oxidase	Urease	Indole	Citrate	ONPG (β-gal)	H₂S	Gelatinase	DNase	Morphology	Gram stain	Blood agar	MacConkey agar	Nutrient agar	Mannitol Salt Agar (MSA)	EMB/ CLED	Sources	Code	No.
+	+	−	+	−	−	−	−	+	+	Cocci	Gram-positive	+	–	+	+	–		S2	1
+	−	−	−	−	+	+	+	−	−	Bacilli	Gram-negative	+	+	+	–	+		S3	2
+	−	−	+	–	+	+	−	−	−	Bacilli	Gram-negative	+	+	+	–	+		S3	3
+	−	−	–	−	–	+	−	−	−	Bacilli	Gram-negative	+	+	+	–	+		S3	4
+	−	+	+	−	+	−	−	−	−	Bacilli	Gram-negative	+	+	+	–	+		S2	5
+	−	+	−	−	−	−	−	−	−	Bacilli	Gram-negative	+	+	+	–	+		S2	6
+	−	−	+	−	+	+	−	−	−	Bacilli	Gram-negative	+	+	+	–	+		S4	7
+	−	−	−	−	+	–	−	+	+	Bacilli	Gram-negative	+	+	+	–	+		S1	8
+	−	+	−	−	+	−	−	−	−	Bacilli	Gram-negative	+	+	+	–	+		S2	9
+	−	−	+	−	−	−	−	−	−	Cocci	Gram-positive	+	+	+	–	+		S1	10

The final identification of bacterial isolates obtained from different samples was performed using the VITEK automated system, Table 2. The results indicate the species-level identification along with the corresponding probability percentage for each isolate. Based on the biochemical profiles as shown in Table 1 and known pathogenic characteristics of the identified species, the bacterial isolates from *Musca demostica* were categorized as either pathogenic or non-pathogenic. The majority of isolates (80%) were identified as pathogenic, including *Staphylococcus pseudintermedius, Citrobacter freundii, Raoultella ornithinolytica, Pantoea agglomerans, Rhizobium radiobacter, Sphingobacterium thalpophilum, Raoultella planticola,* and *Serratia plymuthica*. In contrast, *Sphingomonas paucimobilis* and *Staphylococcus lentus* (20%) were considered non-pathogenic based on their low virulence potential and clinical relevance. These results highlight the microbial diversity across the collected samples and demonstrate the reliability of the VITEK system for accurate bacterial identification and assessment of pathogenicity.

**Table 2:** Identification and pathogenic potential of bacterial isolates based on VITEK analysis.

No.	Code	VITEK result (probability %)	Final identification	Pathogenicity
1	S2	98%	*S. pseudintermedius*	Pathogenic
2	S3	99%	*C. freundii*	Pathogenic
3	S3	97%	*R. ornithinolytica*	Pathogenic
4	S3	95%	*Pantoea agglomerans*	Pathogenic
5	S2	99%	*R. radiobacter*	Pathogenic
6	S2	94%	*S. thalpophilum*	Pathogenic
7	S4	98%	*R. planticola*	Pathogenic
8	S1	96%	*S. plymuthica*	Pathogenic
9	S2	99%	*S. paucimobilis*	Non-pathogenic
10	S1	98%	*S. lentus*	Non-pathogenic

## Discussion

Flies have a short life cycle, high fertility rates, and are strong, quick-adapting organisms. In all environments, they are the most varied insects. The insect ecosystem is being disrupted by global climate fluctuations and human disruption of the agro-environment. We described the distribution and diversity of medical flies in the Sharkia Governorate in our study. Twelve fly species from five families, Calliphoridae, Muscidae, Sarcophagidae, Piophilidae, and Phoridae were found in the Sharkia Governorate, according to the current study. These results are consistent with research [11] and [29], which gathered 12 dipterous species from three distinct sampling locations in Tayma, Saudi Arabia. While 33 different species of flies from the Matruh Governorate, Egypt were captured by [30]. Eleven dipterous species from the Tabuk region were gathered from five locations, according to study [4]. In the present study there was spectacular seasonal variation of *Chrysomya megacephala* with moderate, but constant activity of *Sarcophaga carnaria* might be seen. The absence of *Chrysomya albiceps*, *Lucilia sericata, Piophila casei* speacies during winter even though it was present during the summer. Remarkably, the *Musca domestica* proved to be the most abundant species during all seasons. The abundance of *Musca domestica* species was reported in numerous studies [31–33].

Our research supported the findings of [4], which stated that the genus *Musca* was the most prevalent in livestock facilities, including slaughterhouses and cattle markets. The identical results from Yemen (the southern portion of the Arabian Peninsula) were also reported by [34]. Given that the genus *Musca* is widely distributed around the world and has a high degree of environmental adaptability and acclimatization, this fact is acceptable. Nevertheless, other fly taxa, such as *Chrysomya albiceps, Wohlfahrtia nuba, and Chrysomya bezziana,* were shown to be more prevalent in other Saudi Arabian studies [35]. Study [36] reported that the dominant flies were *Coproica vegans* and *Anatricus erianceus* collected from animal facilities located in the South of Saudi Arabia. During seasonal variations, the muscidae was the most abundant family and much more successful than the other families in all seasons (p < 0.001), while the Calliphoridae was the second most abundant (P < 0.05). The Sarcophagidae, Piophilidae and Phoridae were much fewer in number (P < 0.05) in the present study. These findings match with [4,11,34], While Mismatch with [35,36] applied in Saudi Arabia. The extraordinary variety in the geographical, physical, and chemical variables which may exhibit significant fluctuation among different locations of Saudi Arabia is likely the cause of this disagreement in the findings across various literature. It is well established that, together with humidity and rainfall, temperature is the primary factor influencing dipterous fly diversity and abundance [37,38].

Our study also revealed that Shannon_H values and Simpson_1-D indices showed high levels of diversity all through the year, although with a small drop during winter months. Evenness indices (Equitability_J) reflect distributed resources among species evenly. The summers had the highest values of Fisher and Margalef of alpha. The investigation conducted by [39] and [11] yielded species index values that both confirmed and suggested the species disturbance between locations over the study period. The area is somewhat contaminated, as shown by the species richness index ranging from 0.3338 to 2.149 and the Shannon index ranging from 0.4641 to 0.9341. The evenness index results demonstrated that human activity had an impact on the equitable distribution of flies in the research region. Additionally, [40] discovered that, on a temporal scale, species richness was favorably correlated with total abundance; however, the strength of this correlation was negatively correlated with elevated trophic status. As the trophic level rose, so did insect numbers and species dominance.

Diversity indices give better information about the environmental conditions under which the organisms live than consideration of individual taxa alone [41]. Numerous environmental organizations worldwide have employed biological indicators to track the condition and patterns of aquatic ecosystems [42]. The richness of flies in the studied area is a crucial indicator of both ecological stability and biological state [43]. According to a study [44], the species richness index varies from 1 to 5, indicating moderate contamination; a bigger index denotes a healthier body of water, but a tendency toward 1 suggests increased pollution and damage may be suspected. The results of the current investigation revealed that the bacterial isolates from *Musca demostica* were categorized as either pathogenic or non-pathogenic.

The majority of isolates (80%) were identified as pathogenic, while (20%) were considered non-pathogenic based on their low virulence potential and clinical relevance. Our results are consistent with a study [12] that demonstrated the presence of *Bacillus* species, *Escherichia coli, Pseudomonas aeruginosa, Klebsiella pneumonia, Staphylococcus epidermidis, and Staphylococcus aureus* on the exterior of house flies. Numerous scientists have found that houseflies are powerful carriers of bacteria like *Salmonella spp*. [45], *Campylobacter jejuni* [46], *Staphylococcus aureus, Enterococcus faecalis,* and *Pseudomonas aeruginosa* [47], *Shigella* spp. [48], and *Escherichia coli* [49]. Among the identified isolates, several were classified as pathogenic due to their known association with human infections. *S. pseudintermedius* is an opportunistic pathogen commonly found in animals but can cause zoonotic infections in humans, particularly skin and soft tissue infections, and may exhibit resistance to multiple antibiotics [50]. *C. freundii* is a facultative anaerobic bacterium implicated in urinary tract infections, neonatal meningitis, and bacteremia, particularly in immunocompromised individuals [51]. Similarly, *R. ornithinolytica* and *R. planticola* have emerged as opportunistic pathogens responsible for wound infections, respiratory tract infections, and sepsis [52]. *P. agglomerans*, although primarily plant-associated, can act as a human pathogen leading to septic arthritis, wound infections, and hospital-acquired bacteremia [53]. *R. radiobacter* (formerly *Agrobacterium tumefaciens*) is an opportunistic bacterium often linked to catheter-related bloodstream infections in hospitalized patients [54]. *S. plymuthica* is another opportunistic species that may cause urinary and respiratory tract infections, particularly in immunocompromised hosts [55]. *S. thalpophilum,* though less common, has been reported to cause infections in clinical settings, including septicemia and wound infections [56]. The presence of these pathogenic species in the examined samples suggests possible environmental contamination or cross-transmission sources, highlighting the need for strict hygienic measures and continuous monitoring to prevent potential health risks to humans. In contrast, the non-pathogenic isolates identified in this study, including *S. paucimobilis* and *S. lentus,* are generally considered low risk to human health. *S. paucimobilis* is an environmental bacterium frequently isolated from soil and water; it plays a role in the biodegradation of complex organic compounds. Although it is occasionally detected in clinical specimens, infections are rare and usually occur in immunocompromised individuals [57]. *S. lentus*, a coagulase-negative species, is typically part of the normal skin flora of humans and animals and is generally regarded as non-pathogenic. However, in rare cases, it may cause opportunistic infections under favorable conditions [58]. The detection of these non-pathogenic bacteria indicates normal environmental or commensal microbial presence and provides a useful contrast to the pathogenic strains, helping to differentiate between potential health threats and naturally occurring microorganisms in the studied samples. The behavioral characteristics of the house fly Musca domestica, ensure its contact with food and wastes of man and his animals. In this manner the house flies are able to transport pathogenic organisms from infected materials to human. *Escherichia coli, Staphylococci aureus, Pseudomonas and Proteus spp*. are play role in diarrhea diseases in children. Helicobacter pylori are a major pathogenic factor in gastro duodenal disease, including chronic type B gastritis, duodenal ulcers, and gastric adenoid-carcinoma.

## Conclusion

This study provides a comprehensive longitudinal analysis of medically significant dipteran diversity and seasonal phenology within the Sharkia Governorate. By identifying twelve species across five families, the findings demonstrate that fly abundance is not uniform but is driven by distinct seasonal successions and climatic constraints. Crucially, the presence of stable populations year-round, punctuated by high-density fluctuations in favorable seasons, identifies specific temporal windows of elevated primary transmission risk for fly-borne pathogens. These data transcend basic ecological mapping; they establish a critical baseline for targeted vector surveillance and the development of evidence-based integrated pest management (IPM) strategies in Egypt. To mitigate future public health threats, future research must transition toward the molecular characterization of the microbial “hitchhikers” associated with these taxa. Strengthening the integration of ecological monitoring with microbiological diagnostics will be paramount in constructing a robust early-warning system for fly-borne disease outbreaks in the region.

## Supporting information

S1 Table Median ± SE of the mean for recorded abundance at Faqus. (DOCX)

S2 Table Median ± SE of the mean for recorded abundance at Hehia. (DOCX)

S3 Table Median ± SE of the mean for recorded abundance at Al-Ibrahimia. (DOCX)

S4 Table Median ± SE of the mean for recorded abundance at Minya Al-Qamh. (DOCX)

S5 Table Environmental indices of the investigated sites. (DOCX)
