# Supplementary material for: Distribution and seasonal abundance of medically important flies in Sharkia Governorate, Egypt and their associated bacteria
Source: PLoS One. 2026 May 4;21(5):e0348022. doi: 10.1371/journal.pone.0348022 (PMC13138619; doi:10.1371/journal.pone.0348022)
Supplement: S1 Table — (DOCX) [file pone.0348022.s001.docx]

**Table (S1):** Median± SE of mean for recorded abundance at Faqus

| Season | Families | Sp. | Faqus | | | |
| --- | --- | --- | --- | --- | --- | --- |
|  |  |  | **Al-Nahaseen** | **Al-Samanh** | **AL-Fadadnh** | **Al-Gaafrh** |
| Summer | Calliphoridae | *Chrysomya albicep* | 2.5±0.85 | 3.5±1.75 | 3±1.49 | 1.5±1.08 |
|  |  | *Chrysomya megacephala* | 24.5±0.65 | 24.5±0.65 | 22.5±0.85 | 26.5±1.31 |
|  |  | *Lucilia sericata* | 5.5±0.65 | 5.5±1.04 | 4±0.58 | 7.5±0.85 |
|  |  | *Calliphora vicina* | 18.5±0.65 | 21.5±0.85 | 21±1.03 | 20.75 |
|  |  | *Calliphora vomitoria* | 15.5±1.25 | 16.5±0.48 | 15±0.63 | 14±0.41 |
|  | Muscidae | *Musca domestica* | 40±2.25 | 37.5±3.48 | 40.5±2.5 | 40.5±3.42 |
|  |  | *Musca sorbens* | 5.5±0.65 | 5.5±0.85 | 4.5±1.03 | 5±0.75 |
|  |  | *Stomoxys calcitrans* | 12±1.11 | 12±0.63 | 10.91 | 13.5±0.85 |
|  | Sarcophagidae | *Sarcophaga carnaria* | 8.5±0.71 | 8.5±1.8 | 8.5±1.31 | 11±1.49 |
|  |  | *Wohlfartia magnifica* | 8±0.96 | 8.5±0.85 | 9.5±0.65 | 8±0.63 |
|  | Piophilidae | *Piophila casei* | 7.5±1.93 | 8±0.96 | 6.5±0.71 | 6±0.96 |
|  | Phoridae | *Megaselia scalaris* | 5.5±0.65 | 4±1.49 | 6.5±0.85 | 4±2.81 |
| Autumn | Calliphoridae | *Chrysomya albicep* | 2.5±0.85 | 3.5±1.08 | 2.5±0.85 | 3±1.11 |
|  |  | *Chrysomya megacephala* | 19.5±0.65 | 19.5±0.65 | 17.5±0.85 | 21.5±1.31 |
|  |  | *Lucilia sericata* | 3.5±0.71 | 3.5±0.48 | 4±1.18 | 5±1.25 |
|  |  | *Calliphora vicina* | 16.5±0.85 | 18.5±0.65 | 17.5±0.65 | 17.5±1.25 |
|  |  | *Calliphora vomitoria* | 13±1.11 | 12.5±0.48 | 13±0.91 | 13.5±0.48 |
|  | Muscidae | *Musca domestica* | 32.5±1.93 | 31.5±1.04 | 34.5±1.93 | 33±2.25 |
|  |  | *Musca sorbens* | 2.5±1.25 | 3.5±0.48 | 2.5±0.71 | 4±0.25 |
|  |  | *Stomoxys calcitrans* | 8.5±1.31 | 9±0.41 | 8±0.96 | 11±0.5 |
|  | Sarcophagidae | *Sarcophaga carnaria* | 8±0.75 | 8.5±0.85 | 8.5±0.48 | 9±0.75 |
|  |  | *Wohlfartia magnifica* | 6.5±0.65 | 7±1.85 | 9±0.41 | 7.5±1.93 |
|  | Piophilidae | *Piophila casei* | 5.5±1.8 | 5±1.84 | 6±0.63 | 5±1.35 |
|  | Phoridae | *Megaselia scalaris* | 3.5±1.08 | 3±1.03 | 3.5±1.08 | 4±2.31 |
| Winter | Calliphoridae | *Chrysomya albicep* | 0 | 0 | 0 | 0 |
|  |  | *Chrysomya megacephala* | 5.5±1.8 | 5±1.84 | 6±0.63 | 5±1.35 |
|  |  | *Lucilia sericata* | 0 | 0 | 0 | 0 |
|  |  | *Calliphora vicina* | 1.5±0.48 | 0.5±0.48 | 1.5±0.65 | 1±0.96 |
|  |  | *Calliphora vomitoria* | 0.5±0.71 | 1.5±0.48 | 0.25 | 0.5 |
|  | Muscidae | *Musca domestica* | 8.5±1.31 | 9±0.41 | 8±0.96 | 11±0.5 |
|  |  | *Musca sorbens* | 0 | 0.25 | 0 | 0 |
|  |  | *Stomoxys calcitrans* | 0 | 0 | 0 | 0 |
|  | Sarcophagidae | *Sarcophaga carnaria* | 0 | 0 | 0 | 0 |
|  |  | *Wohlfartia magnifica* | 0 | 0 | 0 | 0 |
|  | Piophilidae | *Piophila casei* | 0 | 0 | 0 | 0 |
|  | Phoridae | *Megaselia scalaris* | 0 | 0 | 0 | 0 |
| Spring | Calliphoridae | *Chrysomya albicep* | 0 | 0.5±0.29 | 0 | 0 |
|  |  | *Chrysomya megacephala* | 5.5±0.65 | 4±1.49 | 6.5±0.85 | 4±2.81 |
|  |  | *Lucilia sericata* | 0 | 0.25 | 0 | 0 |
|  |  | *Calliphora vicina* | 1±1.18 | 1.5±0.65 | 1±0.75 | 1.5±0.65 |
|  |  | *Calliphora vomitoria* | 2.5±0.85 | 3±0.63 | 2.5±0.48 | 2.5±0.95 |
|  | Muscidae | *Musca domestica* | 12±1.11 | 12±0.63 | 10.91 | 13.5±0.85 |
|  |  | *Musca sorbens* | 0 | 0.25 | 0.5±0.29 | 0 |
|  |  | *Stomoxys calcitrans* | 0.5±0.48 | 0.5 | 0.25 | 0.5 |
|  | Sarcophagidae | *Sarcophaga carnaria* | 0.25 | 0.5±0.48 | 0 | 0.5 |
|  |  | *Wohlfartia magnifica* | 0 | 0 | 0 | 0 |
|  | Piophilidae | *Piophila casei* | 0 | 0 | 0 | 0 |
|  | Phoridae | *Megaselia scalaris* | 0 | 0 | 0 | 0 |
