# Supplementary material for: Distribution and seasonal abundance of medically important flies in Sharkia Governorate, Egypt and their associated bacteria
Source: PLoS One. 2026 May 4;21(5):e0348022. doi: 10.1371/journal.pone.0348022 (PMC13138619; doi:10.1371/journal.pone.0348022)
Supplement: S2 Table — (DOCX) [file pone.0348022.s002.docx]

**Table (S2):** Median± SE of mean for recorded abundance at Hehia

| Season | Families | Sp. | Hehia | | | |
| --- | --- | --- | --- | --- | --- | --- |
|  |  |  | **Kafr Abo-Hatb** | **Mahdia** | **Al-Ehsania** | **AL-Shebrawean** |
| Summer | Calliphoridae | *Chrysomya albicep* | 1.5±0.65 | 2±1.11 | 0.5 | 3.5±0.85 |
|  |  | *Chrysomya megacephala* | 24±1.29 | 24.5±1.44 | 22±0.41 | 25±0.96 |
|  |  | *Lucilia sericata* | 5.5±1.25 | 5±1.49 | 4±0.58 | 6±1.78 |
|  |  | *Calliphora vicina* | 20±1.85 | 20±1.11 | 22.5±1.38 | 20.91 |
|  |  | *Calliphora vomitoria* | 16.5±0.48 | 15.5±1.19 | 15±0.82 | 13.5±1.08 |
|  | Muscidae | *Musca domestica* | 44.5±1.47 | 41.5±4.02 | 40.5±2.5 | 35.5±2.39 |
|  |  | *Musca sorbens* | 4±0.75 | 4.5±1.47 | 3.5±1.25 | 4.5±0.85 |
|  |  | *Stomoxys calcitrans* | 11.5±1.04 | 12±0.87 | 9±0.63 | 14±1.65 |
|  | Sarcophagidae | *Sarcophaga carnaria* | 11.5±1.04 | 8±1.11 | 8±1.71 | 11±1.49 |
|  |  | *Wohlfartia magnifica* | 9.5±0.65 | 8.5±0.85 | 4±2.81 | 9±2.35 |
|  | Piophilidae | *Piophila casei* | 6.5±0.71 | 6.5±2.02 | 3±1.89 | 7±1.5 |
|  | Phoridae | *Megaselia scalaris* | 7±0.82 | 5±2.29 | 2.5±2.18 | 5±1.66 |
| Autumn | Calliphoridae | *Chrysomya albicep* | 1.5±0.65 | 3±1.03 | 2.5±0.85 | 4.5±0.85 |
|  |  | *Chrysomya megacephala* | 19±1.29 | 21.5±1.44 | 17.5±0.85 | 20.5±0.85 |
|  |  | *Lucilia sericata* | 5.5±0.71 | 4±1.26 | 3±0 | 4.5±1.19 |
|  |  | *Calliphora vicina* | 20±1.85 | 20±1.11 | 22.5±1.38 | 20.91 |
|  |  | *Calliphora vomitoria* | 5.5±3.33 | 12±3.23 | 11.5±3.03 | 11.5±3.2 |
|  | Muscidae | *Musca domestica* | 36.5±2.9 | 31±3.59 | 31.5±1.93 | 27.5±1.7 |
|  |  | *Musca sorbens* | 3±1.03 | 2±1.31 | 3.5±1.25 | 4±0.63 |
|  |  | *Stomoxys calcitrans* | 10.5±0.65 | 8.5±2.56 | 9±0.25 | 8.5±2.42 |
|  | Sarcophagidae | *Sarcophaga carnaria* | 8.5±0.71 | 6.5±1.94 | 8±1.11 | 9±0.75 |
|  |  | *Wohlfartia magnifica* | 8.5±2.18 | 7.5±2.04 | 4±2.31 | 8.5±2.18 |
|  | Piophilidae | *Piophila casei* | 6.5±0.71 | 5±1.66 | 2.5±1.6 | 5±1.35 |
|  | Phoridae | *Megaselia scalaris* | 5.5±1.55 | 5±1.89 | 2.5±1.6 | 5±1.66 |
| Winter | Calliphoridae | *Chrysomya albicep* | 0 | 0 | 0 | 0 |
|  |  | *Chrysomya megacephala* | 6.5±0.71 | 5±1.66 | 2.5±1.6 | 5±1.35 |
|  |  | *Lucilia sericata* | 0 | 0 | 0 | 0 |
|  |  | *Calliphora vicina* | 0.5±0.48 | 1.5±0.87 | 1.5±0.85 | 2±0.63 |
|  |  | *Calliphora vomitoria* | 1.5±0.29 | 1.5±0.65 | 0.25 | 0.5 |
|  | Muscidae | *Musca domestica* | 10.5±0.65 | 8.5±2.56 | 9±0.25 | 8.5±2.42 |
|  |  | *Musca sorbens* | 0 | 0 | 0 | 0 |
|  |  | *Stomoxys calcitrans* | 0 | 0 | 0 | 0 |
|  | Sarcophagidae | *Sarcophaga carnaria* | 0 | 0 | 0 | 0 |
|  |  | *Wohlfartia magnifica* | 0 | 0 | 0 | 0 |
|  | Piophilidae | *Piophila casei* | 0 | 0 | 0 | 0 |
|  | Phoridae | *Megaselia scalaris* | 0 | 0 | 0 | 0 |
| Spring | Calliphoridae | *Chrysomya albicep* | 0 | 0 | 0 | 0 |
|  |  | *Chrysomya megacephala* | 7±0.82 | 5±2.29 | 2.5±2.18 | 5±1.66 |
|  |  | *Lucilia sericata* | 0 | 0 | 0 | 0 |
|  |  | *Calliphora vicina* | 1.5±0.48 | 1.5±0.48 | 1±0.25 | 1.5±0.65 |
|  |  | *Calliphora vomitoria* | 2±0.25 | 4.5±1.31 | 2.5±0.48 | 2.5±0.95 |
|  | Muscidae | *Musca domestica* | 11.5±1.04 | 12±0.87 | 9±0.63 | 14±1.65 |
|  |  | *Musca sorbens* | 0 | 0 | 0 | 0 |
|  |  | *Stomoxys calcitrans* | 0.5±0.71 | 0.75 | 0.5 | 0.5 |
|  | Sarcophagidae | *Sarcophaga carnaria* | 0 | 0.5 | 0 | 0.5±0.48 |
|  |  | *Wohlfartia magnifica* | 0 | 0 | 0 | 0 |
|  | Piophilidae | *Piophila casei* | 0 | 0 | 0 | 0 |
|  | Phoridae | *Megaselia scalaris* | 0.25 | 0 | 0 | 0 |
