# Supplementary material for: Distribution and seasonal abundance of medically important flies in Sharkia Governorate, Egypt and their associated bacteria
Source: PLoS One. 2026 May 4;21(5):e0348022. doi: 10.1371/journal.pone.0348022 (PMC13138619; doi:10.1371/journal.pone.0348022)
Supplement: S3 Table — (DOCX) [file pone.0348022.s003.docx]

**Table (S3):** Median± SE of mean for recorded abundance at **Al-Ibrahimia**

| Season | Families | Sp. | Al-Ibrahimia | | | |
| --- | --- | --- | --- | --- | --- | --- |
|  |  |  | **Kofor Negm** | **Tal-Mohamed** | **Al-Halawat** | **Mubasher** |
| Summer | Calliphoridae | *Chrysomya albicep* | 4±1.11 | 2±1.11 | 1.5±1.03 | 4±1.11 |
|  |  | *Chrysomya megacephala* | 22±1.85 | 25.5±2.12 | 23.5±0.85 | 27.5±0.71 |
|  |  | *Lucilia sericata* | 8±0.41 | 9.5±0.71 | 5±0.58 | 7.5±0.65 |
|  |  | *Calliphora vicina* | 20.5±1.19 | 20.75 | 19±0.63 | 20.5±1.03 |
|  |  | *Calliphora vomitoria* | 15±1.03 | 14.5±1.04 | 14.5±1.25 | 16.5±0.85 |
|  | Muscidae | *Musca domestica* | 42±1.49 | 33.5±2.78 | 39±3.12 | 42.5±2.39 |
|  |  | *Musca sorbens* | 4±0.41 | 4.5±1.03 | 4.5±0.85 | 4±0.87 |
|  |  | *Stomoxys calcitrans* | 13.5±0.85 | 10±1.32 | 10.5±1.25 | 10.75 |
|  | Sarcophagidae | *Sarcophaga carnaria* | 13±0.63 | 6.5±1.19 | 10±2.63 | 12±0.63 |
|  |  | *Wohlfartia magnifica* | 8.5±0.85 | 11.5±0.65 | 10±2.74 | 9±0.63 |
|  | Piophilidae | *Piophila casei* | 6±0.41 | 9±2.72 | 9.5±0.65 | 7±0.96 |
|  | Phoridae | *Megaselia scalaris* | 5±1.41 | 7±2.32 | 2.5±1.6 | 5±1.78 |
| Autumn | Calliphoridae | *Chrysomya albicep* | 0.5±0.71 | 1±0.96 | 1±0.25 | 3±1.03 |
|  |  | *Chrysomya megacephala* | 18±1.35 | 19.5±3.07 | 22.5±0.65 | 22.5±2.5 |
|  |  | *Lucilia sericata* | 5.5±0.65 | 3.5±2.17 | 5±0.58 | 7.5±0.65 |
|  |  | *Calliphora vicina* | 17±0.96 | 20.75 | 19±0.63 | 18.5±0.95 |
|  |  | *Calliphora vomitoria* | 15±2.69 | 11.5±3.15 | 11.5±3.28 | 14±3.7 |
|  | Muscidae | *Musca domestica* | 32±1.49 | 28.5±2.84 | 31.5±2.17 | 27.5±2.18 |
|  |  | *Musca sorbens* | 4±0.41 | 1±0.75 | 4±0.41 | 2.5±1.03 |
|  |  | *Stomoxys calcitrans* | 10.5±0.65 | 8.5±2.56 | 9±2.5 | 9±0.5 |
|  | Sarcophagidae | *Sarcophaga carnaria* | 4.5±2.92 | 6.5±1.19 | 8.5±2.42 | 4±2.81 |
|  |  | *Wohlfartia magnifica* | 7.5±2.04 | 8±2.22 | 7.5±2.04 | 8.5±2.18 |
|  | Piophilidae | *Piophila casei* | 6±0.41 | 3±1.89 | 8±2.1 | 6±0.5 |
|  | Phoridae | *Megaselia scalaris* | 5±1.41 | 3±2.06 | 2.5±1.6 | 5±1.11 |
| Winter | Calliphoridae | *Chrysomya albicep* | 0 | 0 | 0 | 0 |
|  |  | *Chrysomya megacephala* | 6±0.41 | 3±1.89 | 8±2.1 | 6±0.5 |
|  |  | *Lucilia sericata* | 0 | 0 | 0 | 0 |
|  |  | *Calliphora vicina* | 0.5±0.48 | 1±0.75 | 1±0.41 | 1±0.63 |
|  |  | *Calliphora vomitoria* | 1.5±0.29 | 2.5±0.29 | 0.5±0.29 | 1±0.41 |
|  | Muscidae | *Musca domestica* | 10.5±0.65 | 8.5±2.56 | 9±2.5 | 9±0.5 |
|  |  | *Musca sorbens* | 0 | 0 | 0 | 0 |
|  |  | *Stomoxys calcitrans* | 0 | 0 | 0 | 0 |
|  | Sarcophagidae | *Sarcophaga carnaria* | 0 | 0 | 0 | 0 |
|  |  | *Wohlfartia magnifica* | 0 | 0 | 0 | 0 |
|  | Piophilidae | *Piophila casei* | 0 | 0 | 0 | 0 |
|  | Phoridae | *Megaselia scalaris* | 0 | 0 | 0 | 0 |
| Spring | Calliphoridae | *Chrysomya albicep* | 0 | 0 | 0 | 0 |
|  |  | *Chrysomya megacephala* | 6±0.41 | 9±2.72 | 9.5±0.65 | 7±0.96 |
|  |  | *Lucilia sericata* | 0 | 0 | 0 | 0 |
|  |  | *Calliphora vicina* | 1±0.25 | 1±0.25 | 1±0.25 | 2±0.25 |
|  |  | *Calliphora vomitoria* | 2±0.25 | 6±1.89 | 2.5±0.48 | 3.5±0.65 |
|  | Muscidae | *Musca domestica* | 13±0.63 | 6.5±1.19 | 10±2.63 | 12±0.63 |
|  |  | *Musca sorbens* | 0 | 0 | 0 | 0 |
|  |  | *Stomoxys calcitrans* | 0.5 | 0.25 | 0.75 | 1±0.75 |
|  | Sarcophagidae | *Sarcophaga carnaria* | 0 | 0.5 | 0.25 | 0 |
|  |  | *Wohlfartia magnifica* | 0 | 0 | 0 | 0 |
|  | Piophilidae | *Piophila casei* | 0 | 0 | 0 | 0 |
|  | Phoridae | *Megaselia scalaris* | 0 | 0 | 0 | 0 |
