# Supplementary material for: Distribution and seasonal abundance of medically important flies in Sharkia Governorate, Egypt and their associated bacteria
Source: PLoS One. 2026 May 4;21(5):e0348022. doi: 10.1371/journal.pone.0348022 (PMC13138619; doi:10.1371/journal.pone.0348022)
Supplement: S4 Table — (DOCX) [file pone.0348022.s004.docx]

**Table (S4):** Median± SE of mean for recorded abundance at **Minya Al-Qamh**

| Season | Families | Sp. | Minya Al-Qamh | | | |
| --- | --- | --- | --- | --- | --- | --- |
|  |  |  | **Meet Bashar** | **Shubra Alaneb** | **Al-Azezia** | **Bany Helal** |
| Summer | Calliphoridae | *Chrysomya albicep* | 0.5±0.48 | 1.5±0.48 | 1±0.75 | 2.5±0.65 |
|  |  | *Chrysomya megacephala* | 18±1.32 | 17.5±1.08 | 17.5±0.95 | 17.5±2.04 |
|  |  | *Lucilia sericata* | 4.5±1.58 | 6.5±0.65 | 7.5±2.04 | 6.5±1.8 |
|  |  | *Calliphora vicina* | 17.5±0.65 | 16±0.96 | 15.5±1.08 | 14.5±0.65 |
|  |  | *Calliphora vomitoria* | 11.5±0.65 | 13.5±1.04 | 14±0.91 | 10.5±1.55 |
|  | Muscidae | *Musca domestica* | 31.5±1.08 | 37±3.25 | 32±1.89 | 31±2.38 |
|  |  | *Musca sorbens* | 6±0.87 | 4.5±1.31 | 4±1.26 | 2.5±0.85 |
|  |  | *Stomoxys calcitrans* | 12±0.87 | 11±1.03 | 10.5±1.08 | 12±1.26 |
|  | Sarcophagidae | *Sarcophaga carnaria* | 10±3.17 | 12±0.75 | 9.5±2.53 | 8.5±3.68 |
|  |  | *Wohlfartia magnifica* | 7.5±0.65 | 4±2.46 | 7±1.97 | 4.5±2.75 |
|  | Piophilidae | *Piophila casei* | 8±2.1 | 4±2.46 | 7±1.97 | 4±2.31 |
|  | Phoridae | *Megaselia scalaris* | 6.5±1.08 | 2.5±2.18 | 4.5±1.65 | 3±1.89 |
| Autumn | Calliphoridae | *Chrysomya albicep* | 2.5±0.85 | 2.5±0.85 | 2±1.15 | 1±0.75 |
|  |  | *Chrysomya megacephala* | 18±1.32 | 17.5±1.08 | 16±4.21 | 12.5±3.8 |
|  |  | *Lucilia sericata* | 4.5±1.58 | 5.5±1.55 | 3.5±2.35 | 5.5±1.8 |
|  |  | *Calliphora vicina* | 17.5±0.65 | 15±3.94 | 13.5±3.81 | 14±3.72 |
|  |  | *Calliphora vomitoria* | 11.5±0.65 | 11.5±0.48 | 6±3.61 | 9±2.35 |
|  | Muscidae | *Musca domestica* | 31±2.78 | 30.5±1.75 | 30±3.12 | 22±1.65 |
|  |  | *Musca sorbens* | 3.5±1.25 | 2±1.31 | 3.5±0.95 | 2.5±0.85 |
|  |  | *Stomoxys calcitrans* | 9.5±2.6 | 9.5±2.6 | 9±2.46 | 8.5±2.42 |
|  | Sarcophagidae | *Sarcophaga carnaria* | 10±2.63 | 12±0.75 | 4.5±2.92 | 6.5±1.8 |
|  |  | *Wohlfartia magnifica* | 7.5±0.65 | 4±2.46 | 3.5±2.02 | 4±2.46 |
|  | Piophilidae | *Piophila casei* | 8±2.1 | 4±2.46 | 6.5±1.68 | 3±2.06 |
|  | Phoridae | *Megaselia scalaris* | 4.5±1.58 | 2.5±2.18 | 2±1.31 | 1±1.65 |
| Winter | Calliphoridae | *Chrysomya albicep* | 0 | 0 | 0 | 0 |
|  |  | *Chrysomya megacephala* | 8±2.1 | 4±2.46 | 6.5±1.68 | 3±2.06 |
|  |  | *Lucilia sericata* | 0 | 0 | 0 | 0 |
|  |  | *Calliphora vicina* | 0.5±0.71 | 0.5±0.48 | 1±0.75 | 1.5±0.29 |
|  |  | *Calliphora vomitoria* | 1±0.41 | 2±0.41 | 1±0.41 | 1±0.41 |
|  | Muscidae | *Musca domestica* | 9.5±2.6 | 9.5±2.6 | 9±2.46 | 8.5±2.42 |
|  |  | *Musca sorbens* | 0 | 0 | 0 | 0 |
|  |  | *Stomoxys calcitrans* | 0 | 0 | 0 | 0 |
|  | Sarcophagidae | *Sarcophaga carnaria* | 0 | 0 | 0 | 0 |
|  |  | *Wohlfartia magnifica* | 0 | 0 | 0 | 0 |
|  | Piophilidae | *Piophila casei* | 0 | 0 | 0 | 0 |
|  | Phoridae | *Megaselia scalaris* | 0 | 0 | 0 | 0 |
| Spring | Calliphoridae | *Chrysomya albicep* | 0 | 0 | 0 | 0 |
|  |  | *Chrysomya megacephala* | 8±2.1 | 4±2.46 | 7±1.97 | 4±2.31 |
|  |  | *Lucilia sericata* | 0 | 0 | 1±0.25 | 1±0.25 |
|  |  | *Calliphora vicina* | 1.5±0.29 | 2.5±0.65 | 2±0.25 | 3±0.25 |
|  |  | *Calliphora vomitoria* | 7.5±2.17 | 6.5±1.31 | 5.5±0.48 | 6±1.85 |
|  | Muscidae | *Musca domestica* | 10±3.17 | 12±0.75 | 9.5±2.53 | 8.5±3.68 |
|  |  | *Musca sorbens* | 0 | 1±0.41 | 0.5±0.29 | 1±0.41 |
|  |  | *Stomoxys calcitrans* | 0.5 | 1.5±0.48 | 1±0.41 | 0.5±0.29 |
|  | Sarcophagidae | *Sarcophaga carnaria* | 0.5 | 0.5±0.48 | 0.25 | 0.25 |
|  |  | *Wohlfartia magnifica* | 0 | 0 | 0 | 0 |
|  | Piophilidae | *Piophila casei* | 0 | 0 | 0 | 0 |
|  | Phoridae | *Megaselia scalaris* | 0 | 0 | 0 | 0 |
