# Supplementary material for: Distribution and seasonal abundance of medically important flies in Sharkia Governorate, Egypt and their associated bacteria
Source: PLoS One. 2026 May 4;21(5):e0348022. doi: 10.1371/journal.pone.0348022 (PMC13138619; doi:10.1371/journal.pone.0348022)
Supplement: S5 Table — (DOCX) [file pone.0348022.s005.docx]

**Table (S5):** Environmental indices of investigated sites

| Season | Environmental indices | Faqus | | | | Hehia | | | | Al-Ibrahimia | | | | Minya Al-Qamh | | | |
| --- | --- | --- | --- | --- | --- | --- | --- | --- | --- | --- | --- | --- | --- | --- | --- | --- | --- |
|  |  | **Al-Nahaseen** | **Al-Samanh** | **AL-Fadadnh** | **Al-Gaafrh** | **Kafr Abo-Hatb** | **Mahdia** | **Al-Ehsania** | **AL-Shebrawean** | **Kofor Negm** | **Tal-Mohamed** | **Al-Halawat** | **Mubasher** | **Meet Bashar** | **Shubra Alaneb** | **Al-Azezia** | **Bany Helal** |
| Summer | Dominance_D | 0.14 ± 0.01 | 0.13 ± 0.01 | 0.14 ± 0.01 | 0.14 ± 0.02 | 0.14 ± 0.01 | 0.15 ± 0.01 | 0.17 ± 0.02 | 0.14 ± 0.02 | 0.13 ± 0.01 | 0.13 ± 0.02 | 0.15 ± 0.02 | 0.13 ± 0.01 | 0.13 ± 0.02 | 0.15 ± 0.02 | 0.14 ± 0.02 | 0.15 ± 0.03 |
|  | Simpson_1-D | 0.87 ± 0.01 | 0.88 ± 0.01 | 0.87 ± 0.01 | 0.87 ± 0.02 | 0.87 ± 0.01 | 0.86 ± 0.01 | 0.84 ± 0.02 | 0.87 ± 0.02 | 0.88 ± 0.01 | 0.88 ± 0.02 | 0.86 ± 0.02 | 0.88 ± 0.01 | 0.88 ± 0.02 | 0.86 ± 0.02 | 0.87 ± 0.02 | 0.86 ± 0.03 |
|  | Shannon_H | 2.24 ± 0.02 | 2.24 ± 0.05 | 2.24 ± 0.06 | 2.21 ± 0.09 | 2.21 ± 0.03 | 2.16 ± 0.07 | 2.02 ± 0.07 | 2.21 ± 0.11 | 2.25 ± 0.03 | 2.26 ± 0.11 | 2.15 ± 0.11 | 2.25 ± 0.04 | 2.21 ± 0.07 | 2.12 ± 0.12 | 2.15 ± 0.1 | 2.1 ± 0.13 |
|  | Evenness_e^H/S | 0.8 ± 0.04 | 0.82 ± 0.03 | 0.8 ± 0.03 | 0.81 ± 0.05 | 0.78 ± 0.05 | 0.79 ± 0.03 | 0.79 ± 0.03 | 0.81 ± 0.05 | 0.81 ± 0.05 | 0.83 ± 0.03 | 0.82 ± 0.04 | 0.79 ± 0.03 | 0.85 ± 0.02 | 0.84 ± 0.04 | 0.86 ± 0.02 | 0.84 ± 0.03 |
|  | Brillouin | 2.07 ± 0.02 | 2.08 ± 0.04 | 2.07 ± 0.05 | 2.05 ± 0.08 | 2.05 ± 0.03 | 2 ± 0.08 | 1.86 ± 0.06 | 2.04 ± 0.1 | 2.08 ± 0.03 | 2.09 ± 0.09 | 1.99 ± 0.09 | 2.09 ± 0.04 | 2.03 ± 0.07 | 1.95 ± 0.11 | 1.97 ± 0.09 | 1.92 ± 0.12 |
|  | Menhinick | 0.96 ± 0.04 | 0.92 ± 0.05 | 0.95 ± 0.05 | 0.9 ± 0.03 | 0.92 ± 0.04 | 0.89 ± 0.01 | 0.82 ± 0.07 | 0.92 ± 0.07 | 0.93 ± 0.04 | 0.93 ± 0.08 | 0.87 ± 0.09 | 0.94 ± 0.02 | 0.95 ± 0.04 | 0.88 ± 0.09 | 0.9 ± 0.07 | 0.91 ± 0.08 |
|  | Margalef | 2.15 ± 0.09 | 2.08 ± 0.11 | 2.14 ± 0.11 | 2.03 ± 0.09 | 2.11 ± 0.1 | 1.99 ± 0.12 | 1.73 ± 0.13 | 2.05 ± 0.18 | 2.12 ± 0.1 | 2.09 ± 0.2 | 1.91 ± 0.21 | 2.16 ± 0.02 | 2.01 ± 0.1 | 1.85 ± 0.26 | 1.87 ± 0.17 | 1.85 ± 0.19 |
|  | Equitability_J | 0.91 ± 0.02 | 0.92 ± 0.01 | 0.91 ± 0.02 | 0.92 ± 0.03 | 0.9 ± 0.03 | 0.9 ± 0.02 | 0.9 ± 0.02 | 0.92 ± 0.03 | 0.92 ± 0.02 | 0.93 ± 0.02 | 0.92 ± 0.02 | 0.91 ± 0.02 | 0.94 ± 0.02 | 0.93 ± 0.02 | 0.94 ± 0.01 | 0.93 ± 0.02 |
|  | Fisher_alpha | 2.98 ± 0.14 | 2.87 ± 0.18 | 2.97 ± 0.19 | 2.77 ± 0.14 | 2.91 ± 0.15 | 2.72 ± 0.16 | 2.33 ± 0.21 | 2.81 ± 0.3 | 2.91 ± 0.15 | 2.89 ± 0.32 | 2.6 ± 0.35 | 2.98 ± 0.05 | 2.78 ± 0.15 | 2.53 ± 0.4 | 2.56 ± 0.27 | 2.55 ± 0.31 |
|  | Berger-Parker | 0.26 ± 0.03 | 0.24 ± 0.04 | 0.27 ± 0.03 | 0.26 ± 0.04 | 0.28 ± 0.03 | 0.28 ± 0.03 | 0.3 ± 0.04 | 0.25 ± 0.04 | 0.26 ± 0.02 | 0.23 ± 0.04 | 0.27 ± 0.04 | 0.25 ± 0.04 | 0.25 ± 0.03 | 0.28 ± 0.02 | 0.27 ± 0.03 | 0.28 ± 0.07 |
| Autumn | Dominance_D | 0.14 ± 0.01 | 0.14 ± 0.02 | 0.14 ± 0.01 | 0.14 ± 0.02 | 0.16 ± 0.02 | 0.16 ± 0.05 | 0.16 ± 0.02 | 0.14 ± 0.02 | 0.14 ± 0.02 | 0.16 ± 0.05 | 0.16 ± 0.04 | 0.14 ± 0.02 | 0.14 ± 0.02 | 0.15 ± 0.03 | 0.18 ± 0.02 | 0.16 ± 0.03 |
|  | Simpson_1-D | 0.87 ± 0.01 | 0.87 ± 0.02 | 0.87 ± 0.01 | 0.87 ± 0.02 | 0.85 ± 0.02 | 0.85 ± 0.05 | 0.85 ± 0.02 | 0.87 ± 0.02 | 0.87 ± 0.02 | 0.85 ± 0.05 | 0.85 ± 0.04 | 0.87 ± 0.02 | 0.87 ± 0.02 | 0.86 ± 0.03 | 0.83 ± 0.02 | 0.85 ± 0.03 |
|  | Shannon_H | 2.17 ± 0.07 | 2.18 ± 0.12 | 2.18 ± 0.09 | 2.17 ± 0.09 | 2.11 ± 0.05 | 2.05 ± 0.27 | 2.05 ± 0.1 | 2.17 ± 0.11 | 2.16 ± 0.09 | 1.99 ± 0.24 | 2.08 ± 0.2 | 2.17 ± 0.07 | 2.16 ± 0.09 | 2.05 ± 0.19 | 1.89 ± 0.12 | 1.97 ± 0.14 |
|  | Evenness_e^H/S | 0.8 ± 0.05 | 0.81 ± 0.03 | 0.81 ± 0.06 | 0.84 ± 0.03 | 0.79 ± 0.08 | 0.81 ± 0.05 | 0.8 ± 0.03 | 0.83 ± 0.02 | 0.83 ± 0.04 | 0.85 ± 0.08 | 0.79 ± 0.05 | 0.84 ± 0.03 | 0.86 ± 0.04 | 0.87 ± 0.02 | 0.86 ± 0.04 | 0.88 ± 0.03 |
|  | Brillouin | 1.98 ± 0.06 | 1.99 ± 0.11 | 1.99 ± 0.08 | 1.99 ± 0.08 | 1.94 ± 0.05 | 1.87 ± 0.25 | 1.87 ± 0.09 | 1.98 ± 0.11 | 1.98 ± 0.08 | 1.83 ± 0.21 | 1.9 ± 0.19 | 1.99 ± 0.07 | 1.98 ± 0.09 | 1.87 ± 0.18 | 1.71 ± 0.11 | 1.77 ± 0.12 |
|  | Menhinick | 1.01 ± 0.12 | 0.99 ± 0.09 | 1 ± 0.1 | 0.92 ± 0.04 | 0.93 ± 0.05 | 0.89 ± 0.11 | 0.92 ± 0.08 | 0.96 ± 0.05 | 0.94 ± 0.07 | 0.83 ± 0.16 | 0.93 ± 0.11 | 0.94 ± 0.04 | 0.94 ± 0.09 | 0.85 ± 0.12 | 0.83 ± 0.07 | 0.92 ± 0.12 |
|  | Margalef | 2.1 ± 0.25 | 2.08 ± 0.26 | 2.08 ± 0.27 | 1.96 ± 0.11 | 1.96 ± 0.12 | 1.83 ± 0.36 | 1.85 ± 0.19 | 1.98 ± 0.17 | 1.97 ± 0.13 | 1.65 ± 0.36 | 1.93 ± 0.28 | 1.97 ± 0.1 | 1.94 ± 0.24 | 1.69 ± 0.34 | 1.51 ± 0.18 | 1.65 ± 0.27 |
|  | Equitability_J | 0.91 ± 0.02 | 0.92 ± 0.01 | 0.92 ± 0.03 | 0.93 ± 0.02 | 0.9 ± 0.04 | 0.91 ± 0.04 | 0.9 ± 0.02 | 0.93 ± 0.01 | 0.92 ± 0.02 | 0.93 ± 0.05 | 0.9 ± 0.03 | 0.93 ± 0.02 | 0.94 ± 0.02 | 0.94 ± 0.01 | 0.93 ± 0.02 | 0.94 ± 0.02 |
|  | Fisher_alpha | 2.97 ± 0.44 | 2.92 ± 0.42 | 2.94 ± 0.45 | 2.7 ± 0.17 | 2.71 ± 0.19 | 2.51 ± 0.54 | 2.56 ± 0.31 | 2.77 ± 0.27 | 2.74 ± 0.23 | 2.24 ± 0.56 | 2.67 ± 0.45 | 2.73 ± 0.16 | 2.69 ± 0.38 | 2.31 ± 0.51 | 2.05 ± 0.27 | 2.31 ± 0.44 |
|  | Berger-Parker | 0.26 ± 0.03 | 0.26 ± 0.02 | 0.27 ± 0.02 | 0.26 ± 0.05 | 0.29 ± 0.04 | 0.27 ± 0.05 | 0.28 ± 0.04 | 0.24 ± 0.04 | 0.26 ± 0.03 | 0.26 ± 0.06 | 0.27 ± 0.04 | 0.24 ± 0.04 | 0.25 ± 0.04 | 0.28 ± 0.03 | 0.31 ± 0.05 | 0.28 ± 0.06 |
| Winter | Dominance_D | 0.46 ± 0.2 | 0.46 ± 0.16 | 0.38 ± 0.06 | 0.51 ± 0.09 | 0.39 ± 0.06 | 0.46 ± 0.11 | 0.62 ± 0.34 | 0.51 ± 0.19 | 0.4 ± 0.05 | 0.46 ± 0.19 | 0.57 ± 0.22 | 0.39 ± 0.08 | 0.62 ± 0.34 | 0.56 ± 0.2 | 0.47 ± 0.08 | 0.57 ± 0.2 |
|  | Simpson_1-D | 0.55 ± 0.2 | 0.55 ± 0.16 | 0.63 ± 0.06 | 0.5 ± 0.09 | 0.62 ± 0.06 | 0.55 ± 0.11 | 0.39 ± 0.34 | 0.5 ± 0.19 | 0.61 ± 0.05 | 0.55 ± 0.19 | 0.44 ± 0.22 | 0.62 ± 0.08 | 0.39 ± 0.34 | 0.45 ± 0.2 | 0.54 ± 0.08 | 0.44 ± 0.2 |
|  | Shannon_H | 0.92 ± 0.35 | 0.95 ± 0.4 | 1.02 ± 0.11 | 0.79 ± 0.21 | 1.06 ± 0.16 | 0.88 ± 0.26 | 0.68 ± 0.59 | 0.8 ± 0.38 | 1.05 ± 0.15 | 0.91 ± 0.31 | 0.73 ± 0.35 | 1.06 ± 0.19 | 0.7 ± 0.6 | 0.78 ± 0.31 | 0.88 ± 0.14 | 0.8 ± 0.36 |
|  | Evenness_e^H/S | 0.86 ± 0.09 | 0.87 ± 0.09 | 0.93 ± 0.1 | 0.9 ± 0.04 | 0.83 ± 0.09 | 0.92 ± 0.1 | 0.89 ± 0.12 | 0.94 ± 0.1 | 0.83 ± 0.08 | 0.92 ± 0.09 | 0.79 ± 0.09 | 0.84 ± 0.06 | 0.85 ± 0.14 | 0.81 ± 0.11 | 0.89 ± 0.12 | 0.77 ± 0.08 |
|  | Brillouin | 0.7 ± 0.28 | 0.71 ± 0.3 | 0.77 ± 0.08 | 0.63 ± 0.16 | 0.82 ± 0.12 | 0.67 ± 0.23 | 0.51 ± 0.47 | 0.6 ± 0.31 | 0.81 ± 0.11 | 0.67 ± 0.24 | 0.54 ± 0.3 | 0.82 ± 0.16 | 0.53 ± 0.49 | 0.57 ± 0.26 | 0.65 ± 0.11 | 0.56 ± 0.29 |
|  | Menhinick | 0.75 ± 0.16 | 0.81 ± 0.3 | 0.78 ± 0.05 | 0.6 ± 0.12 | 0.79 ± 0.13 | 0.72 ± 0.13 | 0.66 ± 0.26 | 0.7 ± 0.2 | 0.81 ± 0.13 | 0.75 ± 0.18 | 0.71 ± 0.13 | 0.82 ± 0.12 | 0.68 ± 0.27 | 0.73 ± 0.13 | 0.75 ± 0.21 | 0.83 ± 0.15 |
|  | Margalef | 0.72 ± 0.26 | 0.8 ± 0.49 | 0.75 ± 0.04 | 0.53 ± 0.19 | 0.84 ± 0.2 | 0.65 ± 0.27 | 0.55 ± 0.44 | 0.58 ± 0.32 | 0.86 ± 0.19 | 0.68 ± 0.21 | 0.64 ± 0.28 | 0.87 ± 0.19 | 0.61 ± 0.48 | 0.66 ± 0.18 | 0.68 ± 0.23 | 0.78 ± 0.26 |
|  | Equitability_J | 0.84 ± 0.16 | 0.87 ± 0.08 | 0.93 ± 0.1 | 0.89 ± 0.01 | 0.85 ± 0.08 | 0.92 ± 0.1 | 0.8 ± 0.23 | 0.91 ± 0.16 | 0.85 ± 0.07 | 0.89 ± 0.16 | 0.73 ± 0.17 | 0.85 ± 0.06 | 0.77 ± 0.19 | 0.75 ± 0.2 | 0.89 ± 0.13 | 0.72 ± 0.15 |
|  | Fisher_alpha | 1.1 ± 0.36 | 1.29 ± 0.77 | 1.14 ± 0.08 | 0.81 ± 0.25 | 1.25 ± 0.3 | 1.01 ± 0.34 | 0.93 ± 0.55 | 0.96 ± 0.46 | 1.29 ± 0.3 | 1.07 ± 0.35 | 0.99 ± 0.36 | 1.3 ± 0.3 | 1 ± 0.58 | 1.03 ± 0.26 | 1.09 ± 0.4 | 1.24 ± 0.38 |
|  | Berger-Parker | 0.59 ± 0.2 | 0.6 ± 0.17 | 0.52 ± 0.06 | 0.67 ± 0.08 | 0.54 ± 0.05 | 0.6 ± 0.13 | 0.72 ± 0.28 | 0.66 ± 0.18 | 0.57 ± 0.04 | 0.63 ± 0.17 | 0.67 ± 0.23 | 0.53 ± 0.09 | 0.7 ± 0.3 | 0.7 ± 0.19 | 0.62 ± 0.11 | 0.73 ± 0.19 |
| Spring | Dominance_D | 0.36 ± 0.09 | 0.34 ± 0.06 | 0.3 ± 0.03 | 0.45 ± 0.19 | 0.33 ± 0.06 | 0.4 ± 0.14 | 0.47 ± 0.19 | 0.38 ± 0.11 | 0.39 ± 0.05 | 0.33 ± 0.04 | 0.4 ± 0.13 | 0.29 ± 0.04 | 0.35 ± 0.11 | 0.29 ± 0.07 | 0.28 ± 0.07 | 0.26 ± 0.07 |
|  | Simpson_1-D | 0.65 ± 0.09 | 0.67 ± 0.06 | 0.71 ± 0.03 | 0.56 ± 0.19 | 0.68 ± 0.06 | 0.61 ± 0.14 | 0.54 ± 0.19 | 0.63 ± 0.11 | 0.62 ± 0.05 | 0.68 ± 0.04 | 0.61 ± 0.13 | 0.72 ± 0.04 | 0.66 ± 0.11 | 0.72 ± 0.07 | 0.73 ± 0.07 | 0.75 ± 0.07 |
|  | Shannon_H | 1.22 ± 0.25 | 1.35 ± 0.22 | 1.37 ± 0.16 | 1.04 ± 0.36 | 1.32 ± 0.2 | 1.1 ± 0.41 | 1 ± 0.42 | 1.21 ± 0.22 | 1.17 ± 0.13 | 1.23 ± 0.14 | 1.12 ± 0.28 | 1.37 ± 0.13 | 1.19 ± 0.27 | 1.45 ± 0.24 | 1.5 ± 0.2 | 1.53 ± 0.21 |
|  | Evenness_e^H/S | 0.82 ± 0.13 | 0.75 ± 0.04 | 0.84 ± 0.04 | 0.78 ± 0.11 | 0.8 ± 0.05 | 0.87 ± 0.08 | 0.82 ± 0.05 | 0.79 ± 0.09 | 0.76 ± 0.02 | 0.87 ± 0.08 | 0.78 ± 0.07 | 0.88 ± 0.04 | 0.84 ± 0.1 | 0.8 ± 0.05 | 0.79 ± 0.05 | 0.84 ± 0.17 |
|  | Brillouin | 0.97 ± 0.21 | 1.03 ± 0.17 | 1.05 ± 0.08 | 0.82 ± 0.34 | 1.03 ± 0.14 | 0.89 ± 0.34 | 0.75 ± 0.36 | 0.94 ± 0.18 | 0.91 ± 0.12 | 0.96 ± 0.1 | 0.87 ± 0.25 | 1.1 ± 0.1 | 0.97 ± 0.25 | 1.17 ± 0.2 | 1.14 ± 0.19 | 1.15 ± 0.25 |
|  | Menhinick | 0.88 ± 0.14 | 1.09 ± 0.18 | 1.04 ± 0.28 | 0.78 ± 0.08 | 0.98 ± 0.21 | 0.75 ± 0.25 | 0.85 ± 0.18 | 0.89 ± 0.14 | 0.89 ± 0.05 | 0.89 ± 0.23 | 0.87 ± 0.11 | 0.89 ± 0.13 | 0.8 ± 0.13 | 1.02 ± 0.2 | 1.2 ± 0.12 | 1.22 ± 0.18 |
|  | Margalef | 1.03 ± 0.27 | 1.35 ± 0.33 | 1.23 ± 0.37 | 0.87 ± 0.22 | 1.19 ± 0.31 | 0.85 ± 0.47 | 0.88 ± 0.36 | 1.04 ± 0.18 | 1.04 ± 0.12 | 1 ± 0.3 | 0.98 ± 0.22 | 1.08 ± 0.19 | 0.93 ± 0.22 | 1.34 ± 0.35 | 1.52 ± 0.22 | 1.54 ± 0.23 |
|  | Equitability_J | 0.86 ± 0.11 | 0.83 ± 0.03 | 0.89 ± 0.02 | 0.79 ± 0.16 | 0.86 ± 0.04 | 0.89 ± 0.07 | 0.82 ± 0.09 | 0.83 ± 0.09 | 0.81 ± 0.03 | 0.91 ± 0.05 | 0.81 ± 0.09 | 0.91 ± 0.03 | 0.87 ± 0.1 | 0.86 ± 0.04 | 0.86 ± 0.05 | 0.9 ± 0.11 |
|  | Fisher_alpha | 1.53 ± 0.41 | 2.14 ± 0.62 | 1.98 ± 0.77 | 1.26 ± 0.29 | 1.83 ± 0.6 | 1.27 ± 0.68 | 1.36 ± 0.51 | 1.55 ± 0.33 | 1.53 ± 0.17 | 1.53 ± 0.54 | 1.47 ± 0.34 | 1.58 ± 0.31 | 1.35 ± 0.33 | 2.04 ± 0.6 | 2.47 ± 0.43 | 2.65 ± 0.56 |
|  | Berger-Parker | 0.54 ± 0.09 | 0.54 ± 0.07 | 0.46 ± 0.02 | 0.61 ± 0.2 | 0.49 ± 0.08 | 0.55 ± 0.15 | 0.63 ± 0.21 | 0.56 ± 0.14 | 0.58 ± 0.04 | 0.47 ± 0.08 | 0.52 ± 0.16 | 0.45 ± 0.05 | 0.47 ± 0.15 | 0.44 ± 0.1 | 0.41 ± 0.09 | 0.41 ± 0.07 |
